# A 16S rRNA gene sequencing based study of oral microbiota in migraine patients in China

**DOI:** 10.1080/21655979.2021.1933840

**Published:** 2021-06-21

**Authors:** Weiqing Jiang, Tingting Wang, Chen Liu, Mingzhu Deng, Xiao Ren, Fei Wang, Yaqing Zhang, Xueying Yu, Lingling Yao, Yonggang Wang

**Affiliations:** a Department of Neurology, Beijing Tiantan Hospital, Capital Medical University, Beijing, China; b Department of Neurology, Shanghai Jiaotong University School of Medicine Affiliated Renji Hospital, Shanghai, China; c Department of Neurology, The First Affiliated Hospital of Zhengzhou University, Zhengzhou, China; d Department of Cardiology, First Affiliated Hospital, Guangdong College of Pharmacy, Guangzhou, China; e Headache Center, China National Clinical Research Center for Neurological Diseases, Beijing, China

**Keywords:** Migraine, oral microbiota, 16s rRNA gene, abundance, functional prediction

## Abstract

Migraine is a primary headache characterized by moderate or severe headache attacks, accompanied with reversible neurological and systemic symptoms. There are rare biomarkers for the disease. While emerging evidence has indicated the connection between gut microbiota and migraine, the relation between oral microbiota and migraine is barely known. Thus, the objective of the current study was to explore a possible correlation between oral microbiota and migraine. We compared the oral microbiota communities of migraine patients (26) with healthy subjects (29) via 16S rRNA gene sequencing. Alpha diversity indices were higher in migraine group compared with control group, whereas beta diversity indices also showed significant difference. A total of 23 genera were found differentially abundant between migraine and control groups. To conclude, there was a significant compositional difference in oral microbiota in migraine patients compared with healthy subjects.

## GRAPHICAL ABSTRACT

**Figure:**
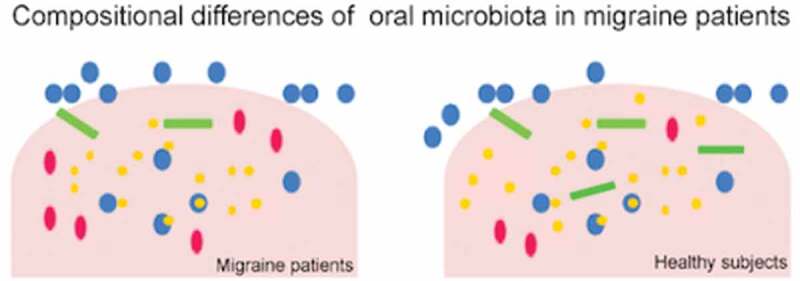

## Introduction

Migraine is a primary headache ranked as the second most disabling neurological disorder in the world [1,2]. It is characterized by moderate or severe headache attacks, accompanied with reversible neurological and systemic symptoms including photophobia, phonophobia, nausea, emesis and cutaneous allodynia [3,4]. The headache attack attributes to activation of the trigeminal sensory pathways which innervate pain-sensitive intracranial structures, including the eye, dura mater, large cerebral and pial blood vessels and the dural venous sinuses [5], whereas cortical spreading depression is considered as the underlying pathophysiological cause of the aura phase [6]. However, there are many controversies and uncertainties in studies on migraine pathogenesis [3]. Moreover, there are rare biomarkers for the disease.

Imbalance of oral microbiota has been connected to a variety of oral diseases such as periodontitis, dental caries and oral mucosal diseases [7], as well as other systemic diseases including bacterial endocarditis [8], ischemic stroke [9], cardiovascular disease [10,11], pancreatic cancer [12], pediatric Crohn’s disease [13], pneumonia [14] and central nervous system (CNS) diseases including Parkinson’s disease [15], Alzheimer’s disease [16] and autism spectrum disorder [17]. However, the relation between oral microbiota and migraine is barely known.

The present study analyzed and compared the oral microbiota composition of migraine patients with healthy subjects via 16S rRNA gene sequencing, so as to explore the relation between oral microbiota and migraine. The study will provide new insight for researchers who are interested in the pathogenesis of migraine.

## Materials and methods

### Study design and subject selection

Protocol of the current study was approved by the Research Ethics Committee, Renji Hospital, Shanghai Jiaotong University School of Medicine (Shanghai, China). Each participant was informed of the objective of the study and signed informed consent. Personal information of each participant was kept confidential.

We designed a cross-sectional study (1 January 2018–18 August 2019) at the outpatient clinic of the Department of Neurology, Renji Hospital, Shanghai Jiaotong University School of Medicine (Shanghai, China).

A cohort of 55 individuals was analyzed. We recruited migraine patients (*n* = 26) diagnosed according to the ICHD-3 (the International Classification of Headache Disorders, 3rd edition) criteria [4]. Healthy participants (*n* = 29) matched by age and sex of the patients were enrolled as the control group. Exclusion criteria for both cases and control participants were any type of CNS disease; serious chronic illnesses including diabetes, hypertension, heart failure and cancers; any type of oral disease; and use of antibiotics or probiotics in the past 3 months. To address potential sources of bias, confounding factors [18] were considered in our exclusion criteria. Meanwhile, to minimize the bias due to diet habits [19], both the patient and control groups were individuals who had lived in the same city (Shanghai) within at least 3 months before sample collection.

## Sample collection and DNA extraction

Saliva samples from migraine patients were collected during outpatient visits. Weight and height were measured. Clinical characteristics of migraine included age of onset, disease course, migraine days (per month), visual analog scale (VAS) scores and medication consumption. Diagnosis was made according to ICHD-3 criteria by neurologists. Subjects were informed not to eat or drink 30 min before saliva collection. Unstimulated saliva was collected with salivettes and was immediately kept on ice and stored in −80°C until use.

Total DNA extraction from the saliva samples was performed with the QIAamp DNA Mini Kit (Qiagen, Germany). DNA quality assessment was conducted via agarose gel electrophoresis.

## 16S rRNA gene sequencing

Polymerase chain reaction (PCR) amplification was performed using universal primers (341 F and 806 R) for the V3-V4 regions of the 16S rRNA gene. Subsequent sequencing was performed on an Illumina Miseq platform for further paired-end reads. Altogether 1,966,711 raw reads and 1,427,897 mapped reads were obtained. Sequence analyses which include operational taxonomic unit (OTU) clustering and taxonomy assignment were performed by Realbio Genomics Institute (China). Detailed protocols were followed as previously described [20].

## Statistical analysis

Statistical analyses were performed using the SPSS ver. 21.0 and R software ver. 3.1.0. Student’s t-test was performed for quantitative data and Pearson’s Chi-square test was performed for categorical data. Alpha diversity and beta diversity indices were calculated with the Qiime program based on the rarefied OTU counts [20]. Distinguishment of the oral microbiota specific to migraine was identified with the linear discriminant analysis (LDA) effect size (LEfSe) method. Differential abundance analyses of migraine and control groups were performed with a generalized linear model with a negative binomial distribution. Prediction of the abundances of functional categories in the Kyoto Encyclopedia of Genes and Genomes (KEGG) ortholog was conducted using Phylogenetic Investigation of Communities by Reconstruction of Unobserved States (PICRUSt). *P* < 0.05 was considered as statistically significant. Detailed analysis methods were followed as previously described [20].

## Results

To explore a possible correlation between oral microbiota and migraine, we compared the oral microbiota communities of migraine patients with healthy subjects via 16S rRNA gene sequencing and found significant compositional differences of oral microbiota in migraine patients compared with healthy subjects. Meanwhile, nitrate-, nitrite- and nitric oxide-reducing bacteria may be correlated with migraine. These results provide implication for the potential relation between oral microbiota and migraine pathogenesis.

## Clinical characteristics

Basic characteristics of migraine and control groups are shown in Table 1. Age, gender and body mass index (BMI) showed no difference between the two groups. The age range of the participants included was from 25 to 58 years old. Migraine patients had average age of onset of 24.5 ± 9.3 years, disease course of 15.8 ± 8.0 years, migraine days (per month) of 10.3 ± 10.0 and VAS scores of 7.4 ± 1.6. Additionally, two patients (7.7%) were diagnosed with migraine with typical aura, five patients (19.2%) were diagnosed with medication-overuse headache and five patients (19.2%) were taking preventive medications (flunarizine, trazodone or deanxit).

**Table 1.** Characteristics of the study subjects

Characteristics	Migraine group (*n* = 26)	Control group (*n* = 29)	*P* value
Age (years)^a^	39.5 (10.6)	39.0 (10.1)	0.879
Female (*n*, %)	22 (84.6%)	24 (82.8%)	0.853
BMI (kg/m^2^)^a^	21.4 (3.2)	22.0 (2.8)	0.432
Age of onset (years)^a^	24.5 (9.3)	-	
Disease course (years)^a^	15.8 (8.0)	-	
Migraine days (per month)^a^	10.3 (10.0)	-	
VAS scores^a^	7.4 (1.6)	-	
With typical aura (*n*, %)	2 (7.7%)	-	
MOH (*n*, %)	5 (19.2%)	-	
Preventive medications (*n*, %)	5 (19.2%)	-	

Data are shown as mean (SD).^a^

BMI: body mass index; VAS: visual analog scale; MOH: medication-overuse headache; SD: standard deviation.

## Species diversity between migraine and control groups

**Figure 1.**
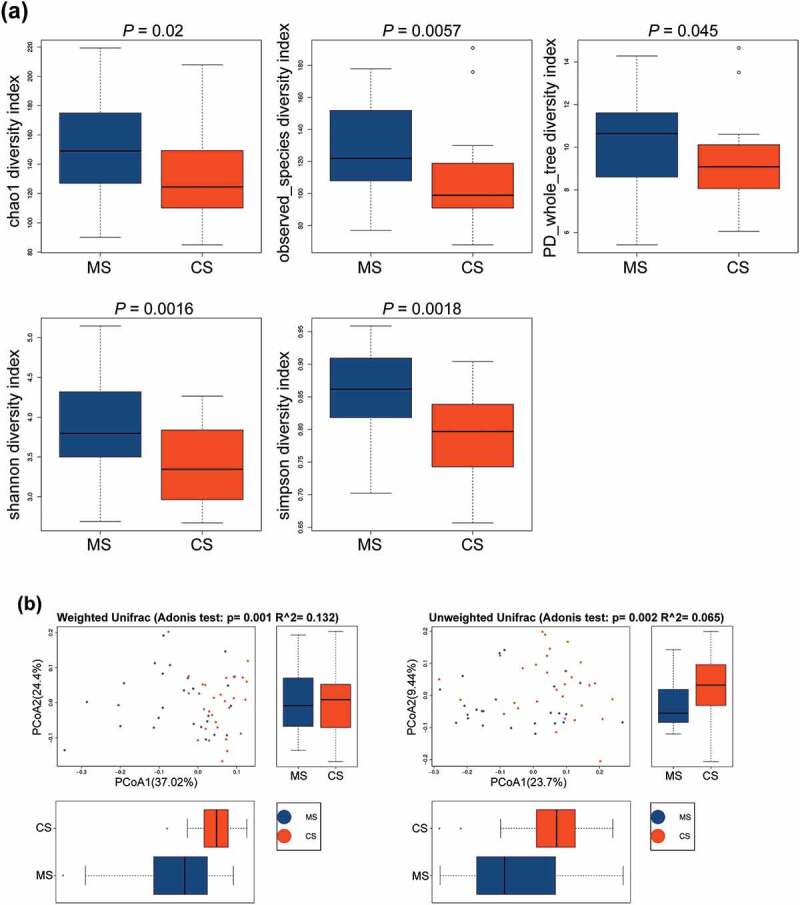
The alpha diversity and beta diversity indices of the oral microbiota in migraine and control groups

Based on OTU levels, alpha diversity indices were analyzed to quantify both ‘species’ richness and diversity [15]. Chao1, observed species, phylogenetic diversity (PD) whole tree, and Shannon and Simpson indices were significantly higher in migraine group than those in control group (Figure 1(a)), indicating that the richness and diversity of the oral microbiota in migraine patients were significantly higher than those in healthy subjects. Meanwhile, beta diversity indices also showed significant differences in weighted and unweighted UniFrac between migraine and control groups (Figure 1(b)), indicating that the composition community [15] of oral microbial in migraine group was different from that in control group.

(a) Differences in alpha diversity indices between migraine and healthy groups measured with Chao 1 index, observed species index, PD whole tree index, Shannon index and Simpson index. From bottom to top, each box plot represents the minimum, first quartile, median, third quartile and maximum values. (b) Differences in beta diversity indices between migraine and healthy groups measured with weighted and unweighted Adonis test and PCOA. The horizontal and vertical axes represent the first and second principal coordinates explaining the greatest proportion of variance to the bacterial communities (showed by percentage). MS: migraine sample group; CS: control sample group; OTU: operational taxonomic unit; PCOA: principal coordinates analysis.

## Taxa alteration between migraine and control groups

**Figure 2.**
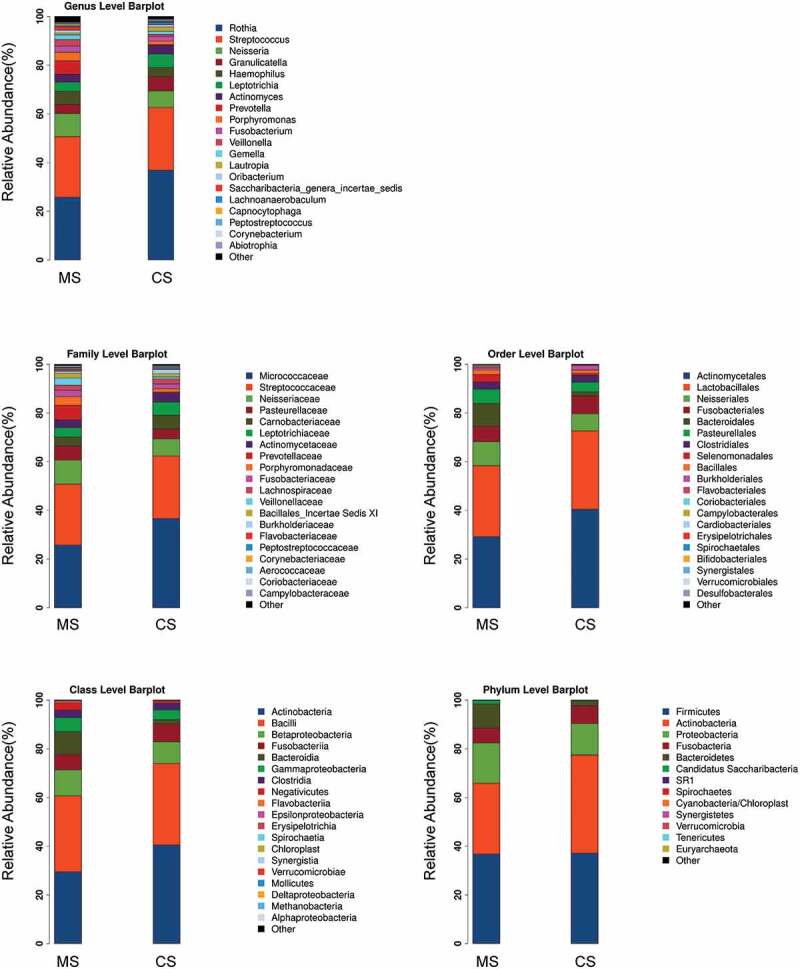
Relative abundances of the oral microbiota in migraine and control groups

**Figure 3.**
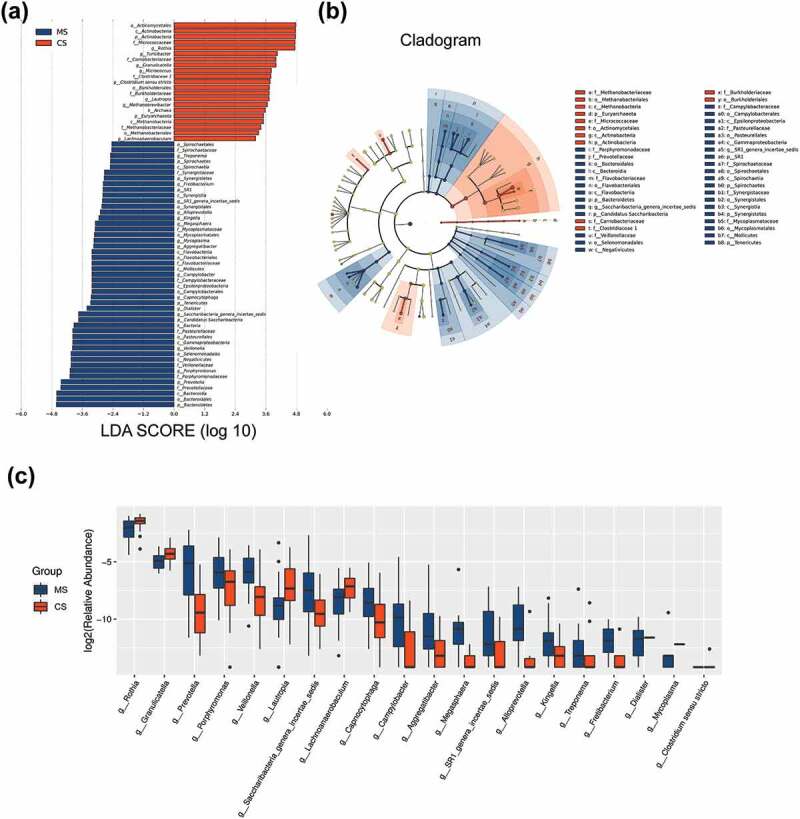
Taxonomic differences of the oral microbiota in migraine and control groups

The data set of this study was composed of 6858 OTUs, covering 208 genera, 228 families, 243 classes, 240 orders and 260 phyla (Figure 2), among which the core microbiota (microbiota covering 100% of the samples) were listed in Table A1. A total of 77 OTUs (data not shown) and 23 genera (Table A2, *P* < 0.05) were found to be distinctively abundant between migraine and control groups. To further identify vital taxonomic differences between migraine and control groups, we conducted LEfSe analysis and found significant abundance differences in oral microbiota between the two groups (Figure 3(a,b), LDA scores (log10) >2, *P* < 0.05). The relative abundances of genera *Rothia, Turicibacter, Granulicatella, Micrococcus, Clostridium sensu stricto, Lautropia, Methanobrevibacter* and *Lachnoanaerobaculum* were higher in control group, while the relative abundances of genera *Treponema, Fretibacterium, SR1 genera incertae sedis, Alloprevotella, Kingella, Megasphaera, Mycoplasma, Aggregatibacter, Campylobacter, Capnocytophaga, Dialister, Saccharibacteria genera incertae sedis, Veillonella, Porphyromonas* and *Prevotella* were higher in migraine group (Figure 3(c), LDA score (log10) >2, *P* < 0.05). These results suggested that the changed abundances of genera were probably associated with migraine.

Barplots showing relative abundances of the oral microbiota at the genus, family, order, class and phylum levels. MS: migraine sample group; CS: control sample group.

(a) Clustering tree showing significant taxonomic differences in the oral microbiota between migraine (negative score) and control (positive score) groups through LEfSe analysis (LDA scores (log10) >2). (b) Cladogram indicating the phylogenetic distribution of the oral microbiota in migraine and control groups through LEfSe analysis (LDA scores (log10) >2). (c) The top 20 differentially abundant genera between migraine and control groups. MS: migraine sample group; CS: control sample group; p: phylum; c: class; o: order; f: family; g: genus; LEfSe: linear discriminant analysis effect size; LDA: linear discriminant analysis.

## Functional predicition

**Figure 4.**
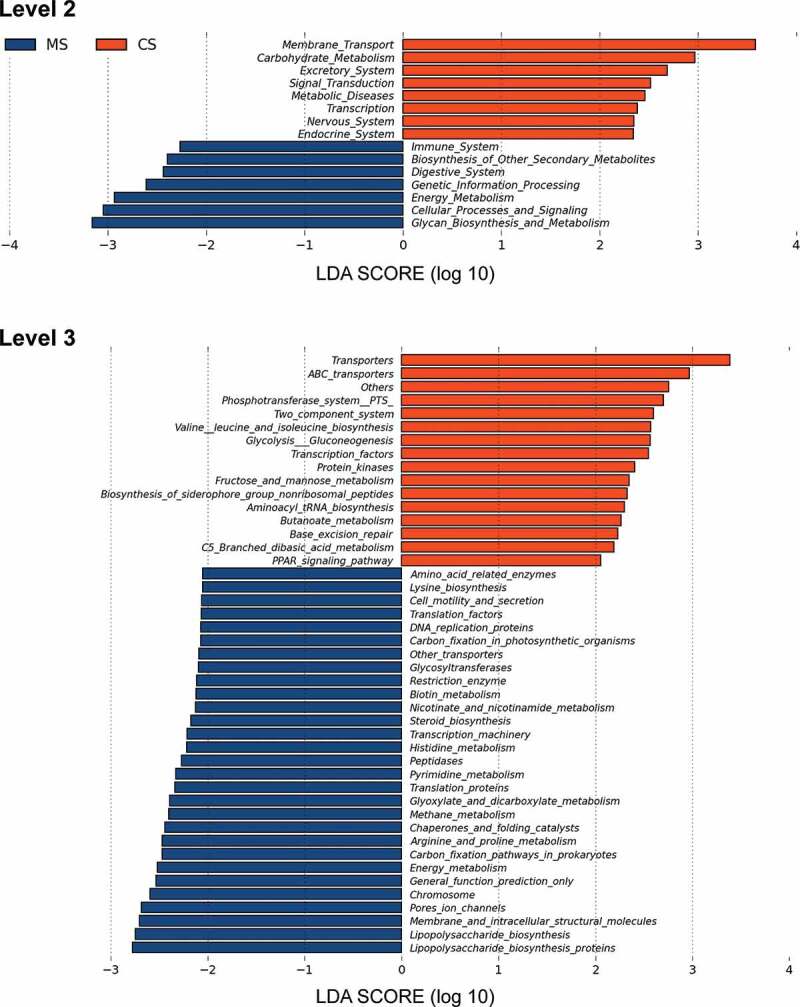
Functional predictions for the oral microbiota of migraine and control groups

We further predicted the abundances of functional categories in the KEGG ortholog (KO) using PICRUSt based on closed-reference OTU. As a result, 73 KOs were identified to be differentially abundant in the oral microbiota between migraine and control groups (Supplementary Figure S1, FDR, *P* < 0.05). To mention microbial gene functions in the level 2 KEGG pathways, pathways involved in membrane transport, carbohydrate metabolism, excretory system, signal transduction, metabolic diseases, transcription, nervous system and endocrine system were higher in the oral microbiota of control group, while the microbial gene functions involving in immune system, biosynthesis of other secondary metabolites, digestive system, genetic information processing, energy metabolism, cellular processes and signaling and glycan biosynthesis and metabolism were higher in the oral microbiota of migraine group (Figure 4, *P* < 0.05).

Level 2 and 3 KEGG pathways significant for the oral microbiota of migraine (negative score) and control (positive score) groups identified using PICRUSt. MS: migraine sample group; CS: control sample group; KEGG: Kyoto Encyclopedia of Genes and Genomes; PICRUSt: Phylogenetic Investigation of Communities by Reconstruction of Unobserved States.

## Discussion

The current study provided evidence of compositional differences in oral microbiota between migraine patients and healthy subjects.

## Oral microbiota and central nervous system

Oral microbial dysbiosis has been reported in CNS diseases including Parkinson’s disease [15], Alzheimer’s disease [16] and autism spectrum disorder [17]. In the past decade, great efforts have been made to illustrate the important role of the gut microbiota in brain function and diseases via the concept of gut microbiota–brain axis [21,22]. Bidirectional communications between the microbiota and brain were suggested to achieve via pathways such as the hypothalamic–pituitary–adrenal (HPA) axis, the neurotransmitter pathways and the immune system and also via recognition of bacterial and host metabolites [23]. While most studies concerned gut microbiota, the impact of oral microbiota on the brain is much less studied.

Nonetheless, emerging studies have implicated that oral microbiota may reach and affect the brain [24]. Oral bacteria including *Treponema* [25] and *C. pneumoniae* [26,27] were detected in postmortem brains of Alzheimer’s disease patients. Meanwhile, higher levels of antibodies against *F. nucleatum, P. intermedia* [28], *A. actinomycetemcomitans, P. gingivalis* and *T. forsythia* [29] were detected in Alzheimer’s disease patients than in healthy subjects. This suggested that some oral bacteria might invade the brain through the brain–blood barrier. Furthermore, endotoxin produced by oral bacteria has been implicated in chronic inflammation and neurodegeneration, especially in Alzheimer’s disease patients [16]. It has been speculated that oral microbiota could reach the brain through direct (via the olfactory tract and olfactory nerve) and indirect (via blood, blood–brain barrier, perivascular spaces and circumventricular organs) mechanisms [17,30]. Meanwhile, oral microbiota are also supposed to affect the brain through inflammation and metabolic alterations [17].

## Oral microbiota and migraine

Previous studies have proved that migraine could be impacted by the gut–brain axis, where the composition of the gut microbiota plays a major role. Though not clearly explained, the underlying mechanism is speculated to be the gut microbiota profile interacting with other multiple factors including inflammatory mediators, neuropeptides, stress hormones and nutritional substances [31]. However, the relation between oral microbiota and migraine is barely known.

Recently, one relevant study detected significantly higher abundances of nitric oxide (NO)-reducing bacteria (genera *Streptococcus* and *Pseudomonas*) in oral samples with self-reported migraine status in the American Gut Project cohort [32]. However, in the current study, abundances of *Streptococcus* and *Pseudomonas* were found to be of no difference between migraine patients and control groups. Indeed, high abundances of *Prevotella* and *Veillonella* and low abundance of *Rothia* were detected in migraine patients in the current study. Interestingly, low abundances of *Prevotella* and *Veillonella* and high abundance of *Rothia* were found to be correlated with greater increases in plasma nitrite in response to nitrate supplementation [33], indicating that these bacteria contribute to NO homeostasis. Moreover, arginine (source of NO) metabolism in the level 3 KEGG pathways was higher in the oral microbiota of migraine group. Taking into consideration that NO is a classic migraine trigger [34] and involves in nociceptive transmission [3] and increased NO stress in migraine patients [35,36], we would propose a potential connection between migraine and these oral bacteria.

Among the 15 genera which were of higher abundance in the migraine group, higher abundances of *Prevotella* and *Veillonella* were also related with oral diseases such as gingivitis, recurrent aphthous ulcer and dental caries [37–39]. However, participants with any type of oral disease were excluded from our study, as listed in the exclusion criteria of our study design. Although clinical oral examination was not conducted, any participant with self-reported oral diseases such as periodontal diseases and dental caries, as well as oral symptoms such as bleeding or swollen gums and oral ulcer, was excluded from our study. Thus, we do not consider the compositional difference of oral microbiota in migraine patients as a result of oral condition.

## Limitations

There are limitations to the current study. First, the sample size of the current study was limited. Second, bias may exist due to the inclusion and exclusion criteria and medication consumption of some patients. Third, as a cross-sectional study, the results were not convincing enough to tell the causal relationship between migraine and oral microbiota dysbiosis. To sum up, it requires larger longitudinal studies focusing on different types of primary headaches to confirm our results.

## Conclusion

The present study is the first to show evidence of compositional differences in oral microbiota in a Chinese cohort of migraine patients compared with healthy subjects. Further and more detailed investigations are expected to verify whether oral dysbacteriosis exists in migraine patients, making oral microbiota a potential biomarker for migraine. Meanwhile, regarding the causal relationship between migraine and oral microbiota dysbiosis, it is worth exploring whether oral microbiota involves in migraine pathogenesis and how oral microbiota could change subsequent to the disease.

## Supplementary Material

Supplemental Material

## Data Availability

The datasets generated and/or analyzed during the current study are available in the [NCBI Sequence Read Archive] repository at [https://www.ncbi.nlm.nih.gov/Traces/study/?acc=PRJNA680860].

## Research highlights

- Compositional differences in oral microbiota in migraine patients
- Nitrate-, nitrite- and nitric oxide-reducing bacteria may be correlated with migraine
- Evidence for migraine epidemiology, pathology and oral microbiology
