# Supplementary material for: A 16S rRNA gene sequencing based study of oral microbiota in migraine patients in China
Source: Bioengineered. 2021 Jun 21;12(1):2523–33. doi: 10.1080/21655979.2021.1933840 (PMC8806455; doi:10.1080/21655979.2021.1933840)
Supplement: Supplemental Material [file KBIE_A_1933840_SM2588.zip › Supplementary/Supplementary materials.docx]

**SUPPLEMENTARY MATERIALS**

**SUPPLEMENTARY TABLES**

Supplementary table

Supplementary table 1.—Core microbiota OTUs

| OTU ID | taxonomy Level | taxonomy Name |
| --- | --- | --- |
| denovo27 | family | Flavobacteriaceae |
| denovo24 | genus | Actinomyces |
| denovo23 | genus | Atopobium |
| denovo21 | genus | Leptotrichia |
| denovo20 | genus | Actinomyces |
| denovo176 | genus | Rothia |
| denovo18 | genus | Veillonella |
| denovo10 | genus | Oribacterium |
| denovo11 | genus | Gemella |
| denovo16 | genus | Lautropia |
| denovo17 | genus | Leptotrichia |
| denovo14 | genus | Rothia |
| denovo15 | genus | Lachnoanaerobaculum |
| denovo146 | genus | Streptococcus |
| denovo72 | genus | Actinomyces |
| denovo180 | order | Lactobacillales |
| denovo227 | class | Bacilli |
| denovo1 | genus | Rothia |
| denovo2 | genus | Streptococcus |
| denovo3 | genus | Granulicatella |
| denovo4 | genus | Fusobacterium |
| denovo5 | genus | Haemophilus |
| denovo7 | genus | Streptococcus |
| denovo9 | genus | Porphyromonas |
| denovo118 | genus | Leptotrichia |

Core microbiota (microbiota covering 100 per cent of the samples) were listed.

OUT, operational taxonomic unit

**Supplementary table** **2.—Genera distinctively abundant between migraine and control groups.**

| taxon name | mean (CS) | mean (MS) | *P* value | FDR |
| --- | --- | --- | --- | --- |
| g__Aggregatibacter | 0.000150966 | 0.00201035 | 0.000370798 | 0.005487817 |
| g__Alloprevotella | 6.44E-05 | 0.000800854 | 0.007469247 | 0.038785283 |
| g__Campylobacter | 0.000259587 | 0.003412871 | 0.000106316 | 0.00262247 |
| g__Capnocytophaga | 0.001870501 | 0.005127521 | 0.003436959 | 0.028259439 |
| g__Clostridium sensu stricto | 1.66E-05 | 2.05E-06 | 0.034967678 | 0.117618554 |
| g__Dialister | 2.21E-05 | 0.000135529 | 0.006107958 | 0.034768375 |
| g__Fretibacterium | 2.76E-05 | 0.000162224 | 0.020734774 | 0.076718664 |
| g__Granulicatella | 0.056155531 | 0.036644215 | 0.002768809 | 0.028259439 |
| g__Kingella | 0.000117827 | 0.00049078 | 0.000212519 | 0.003931603 |
| g__Lachnoanaerobaculum | 0.00795147 | 0.004840034 | 0.009655773 | 0.04203101 |
| g__Lautropia | 0.017316862 | 0.007780607 | 0.004617334 | 0.029191611 |
| g__Megasphaera | 1.10E-05 | 0.000993881 | 0.000658305 | 0.008119095 |
| g__Methanobrevibacter | 2.21E-05 | 0 | 0.01574774 | 0.064740709 |
| g__Micrococcus | 2.03E-05 | 0 | 0.008386007 | 0.038785283 |
| g__Mycoplasma | 7.36E-06 | 7.39E-05 | 0.018703824 | 0.072846472 |
| g__Porphyromonas | 0.013340207 | 0.034032198 | 0.038106535 | 0.122603633 |
| g__Prevotella | 0.003172119 | 0.0539324 | 2.06E-05 | 0.001526886 |
| g__Rothia | 0.361304932 | 0.253078155 | 0.004397795 | 0.029191611 |
| g__SR1_genera_incertae_sedis | 0.000101257 | 0.000872726 | 0.00322785 | 0.028259439 |
| g__Saccharibacteria_genera_incertae_sedis | 0.002457794 | 0.014327077 | 0.004733775 | 0.029191611 |
| g__Treponema | 0.000134396 | 0.000357304 | 0.007869776 | 0.038785283 |
| g__Turicibacter | 1.47E-05 | 2.05E-06 | 0.034967678 | 0.117618554 |
| g__Veillonella | 0.007566692 | 0.025826523 | 7.18E-05 | 0.00262247 |

Name, mean abundance in each group，*P* value of Wilcoxon test, FDR value of the genera distinctively abundant between migraine and control groups are listed.

MS, migraine sample group; CS, control sample group; g, genus; FDR, false discovery rate

**SUPPLEMENTARY FIGURES**


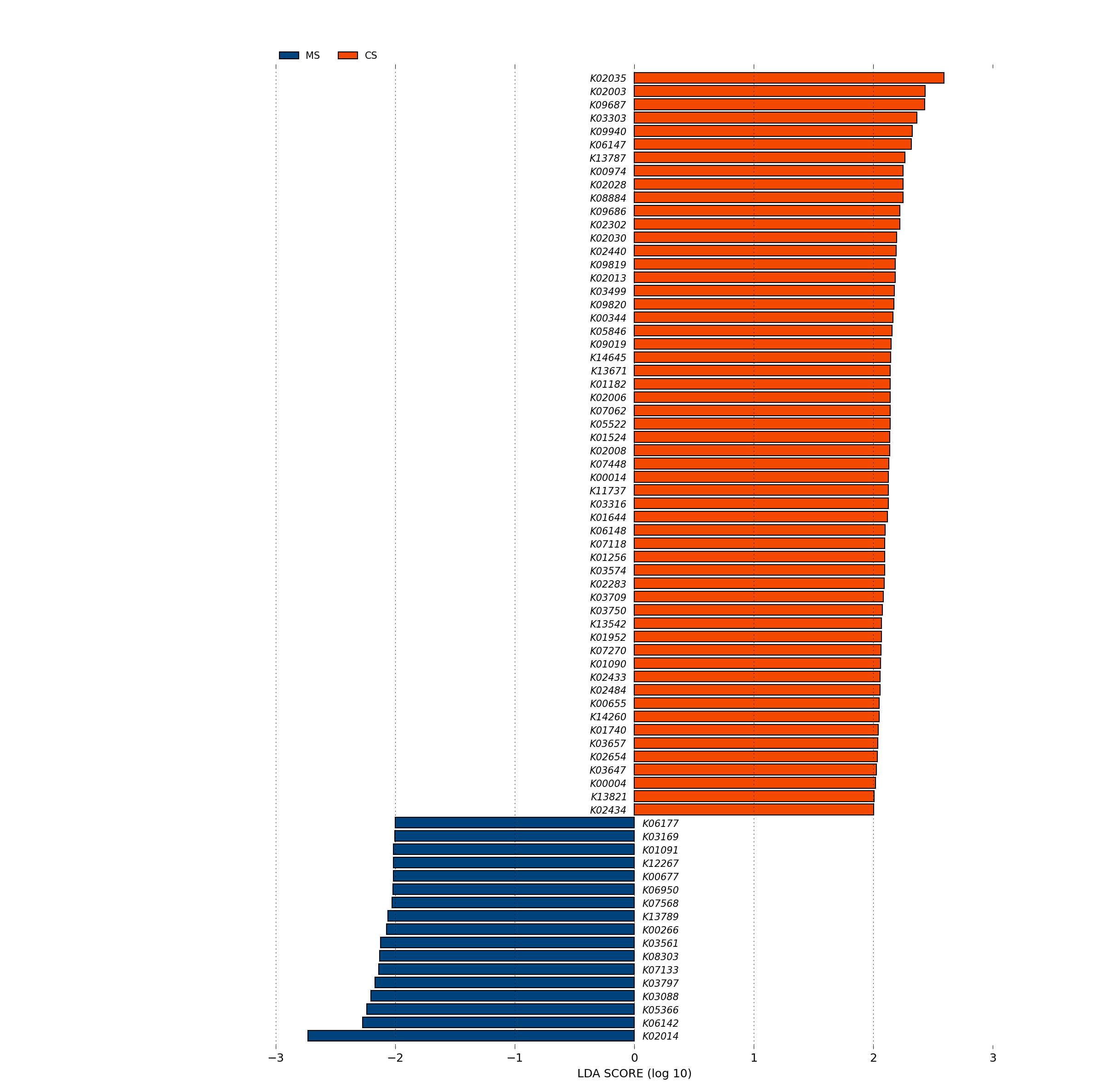


**Supplementary Figure 1.—Functional predictions for the oral microbiota of migraine and control groups.**

The KOs with significantly different abundances in the oral microbiota between migraine (negative score) and control (positive score) groups identified using PICRUSt are shown (FDR, *P* < 0.05 are listed).

MS, migraine sample group; CS, control sample group; KO, Kyoto Encyclopedia of Genes and Genomes ortholog; PICRUSt, Phylogenetic Investigation of Communities by Reconstruction of Unobserved States; FDR, false discovery rate.
